# Treatment of herpes labialis by photodynamic therapy: Study protocol clinical trial (SPIRIT compliant)

**DOI:** 10.1097/MD.0000000000019500

**Published:** 2020-03-20

**Authors:** Andreia La Selva, Renata Matalon Negreiros, Daniela Teixeira Bezerra, Ellen Perin Rosa, Vanessa Christina Santos Pavesi, Ricardo Scarparo Navarro, Marina Stella Bello-Silva, Karen Müller Ramalho, Ana Cecília Corrêa Aranha, Paulo Henrique Braz-Silva, Kristianne Porta Santos Fernandes, Sandra Kalil Bussadori, Anna Carolina Ratto Tempestini Horliana

**Affiliations:** a Postgraduate Program in Biophotonics Applied to Health Sciences, Universidade Nove de Julho, UNINOVE; b Postgraduate Program in Bioengineering and Biomedical Engineering, School of Dentistry, Universidade Brasil; c International Academy of Lasers in Dentistry; d Laser Special Laboratory in Dentistry, LELO, School of Dentistry, University of Sao Paulo; e Post Graduate Program of Dental Sciences, University Ibirapuera; f Department of Dentistry School of Dentistry, University of Sao Paulo; g Division of Pathology, Department of Stomatology, School of Dentistry, University of Sao Paulo; h Laboratory of Virology, Institute of Tropical Medicine of Sao Paulo, University of Sao Paulo, Sao Paulo, Brazil.

**Keywords:** photodynamic therapy, cold sores, herpes simplex, laser

## Abstract

**Background:**

Lesions of herpes labialis are caused by the herpes simplex virus type 1 and cause pain and aesthetic compromise. It is characterized by the formation of small vesicles that coalesce and rupture forming extremely painful ulcers, that evolve to crusts, dry desquamations until their complete remission. Currently the treatment of these lesions is done with acyclovir. Although it diminishes the symptomatology, it causes viral resistance and does not prevent the recurrence of the lesions. It is known that antimicrobial photodynamic therapy (aPDT) has numerous advantages, among them: the reduction of the time of remission, and does not cause resistance. This protocol will determine the effectiveness of PDT in lesions of herpes labialis.

**Materials and methods:**

A total of 30 patients with herpes labialis in the prodromal stage of vesicles, ulcers, and crusts will be selected to participate in the study and randomized into 2 groups: G1 control and G2 experimental. After signing Research Ethics Committee and TA, patients in group G1 will undergo the standard gold treatment for herpes labialis with acyclovir and simulated PDT treatment. Patients in the experimental G2 group will be treated simulating the gold standard treatment of herpes labialis (placebo) and PDT. In all patients, saliva samples will be collected for analysis of cytokines, and will be performed exfoliative cytology in the lesions. The pain will be assessed through a pain scale and a questionnaire of quality of life related to oral health (OHIP-14) will be given to them. Patients will continue to be followed up after 7 days, 1 month, 3 months, and 6 months; if there is a recurrence of the lesion, they will contact the researchers.

Clinical registration: clinicaltrials.gov - NCT 04037475. Registered on July 2019.

## 1 Introduction

The prevalence of herpes labialis increases gradually in childhood, reaching 70% to 80% of adults worldwide.^[1–3]^ Recurrence of herpes occurs in 20% to 40% of the world's population.^[4]^ Herpes labialis are an infectious disease caused by the herpes simplex virus (HSV). HSV type 1 (HSV-1) and HSV-2 subtypes affect the skin and mucous membranes, but the HSV-1 subtype affects the orofacial region more. Recurrent herpes labialis can also be known as “fever blisters” or “cold sores.”^[5]^ Initial primary infections are usually more severe than recurrent ones, since the patient does not have acquired immunity.^[6]^ Herpetic gingivostomatitis usually occurs in young children characterized by painful oro-labial vesicles that coalesce and rupture forming ulcers, lasting between 10 and 14 days, accompanied by fever, malaise, fatigue, pain muscle, and dehydration.^[6–9]^

Clinically, the recurrence of the lesions goes through stages: prodromal, redness, papule, gallbladder, ulcer, crust, dry scaling, and remission of the lesion. Many of these stages may be short or imperceptible.^[6,10,11]^ It is a self-limiting, painful disease that generates discomfort, social restraint, and aesthetic compromise. In immunocompromised patients, recurrent lesions are more aggressive and take longer to heal.^[12,13]^ Because of these factors, associated with several recurrences, studies are important for other alternatives to treat herpes labialis, aiming to a fast resolution of the lesions, with less time of recurrence, without undesirable side effects.

Aciclovir is the most widely used medication to prevent or suppress HSV-1.^[10,14]^ Continuous use of these drugs may develop a high viral resistance.^[7,10]^ Antiviral medications are most effective when the patient is still in the prodromal phase. Aciclovir is effective in reducing the duration and symptoms of infection, but the optimal dose is still unknown.^[9]^ Although acyclovir decreases the duration of symptoms, it does not prevent recurrence.^[15–17]^ In view of the resistance that antiviral treatment can cause and the recurrence of the lesion, some alternative treatments have been studied, such as antimicrobial photodynamic therapy (PDT), which is a good option.^[18]^

The PDT is a therapeutic option in many fields of medicine.^[19]^ It is selective and noninvasive, also indicated for transplant and oncology patients.^[15]^ It consists of the combination of a chromophore agent, such as methylene blue, at a certain concentration, associated with monochromatic laser light at a given wavelength.^[20]^ The photosensitizer (methylene blue) absorbs visible laser light at a suitable wavelength (660 nm) from the ground state to the singlet state, which has a short shelf life. From there, the photosensitizer can lose energy through nonradioactive, radioactive (phosphorescence) processes, or generate reactive oxygen species (ROS) through 2 mechanisms: either by electron transfer, forming free radicals (type I reactions), or through the transfer of energy to molecular oxygen forming singlet oxygen (type II reactions).^[21]^ The lesions of herpes labialis are recurrent in some patients, causing pain, aesthetic commitment, and absenteeism in the work. Your complete remission lasts until 15 days. Treatment with acyclovir decreases painful symptoms; however, it causes viral resistance and does not prevent recurrence of the lesions. It is known that antimicrobial PDT (aPDT) has numerous advantages among them the reduction of the time of remission and, moreover, does not cause resistance; although there are still no well-designed clinical studies that prove its effectiveness, there are only case reports. Therefore, the objective of this study will be to evaluate whether PDT is able to reduce the healing time of these lesions and decrease their recurrence.

## 2 Methods

**Figure 1:**
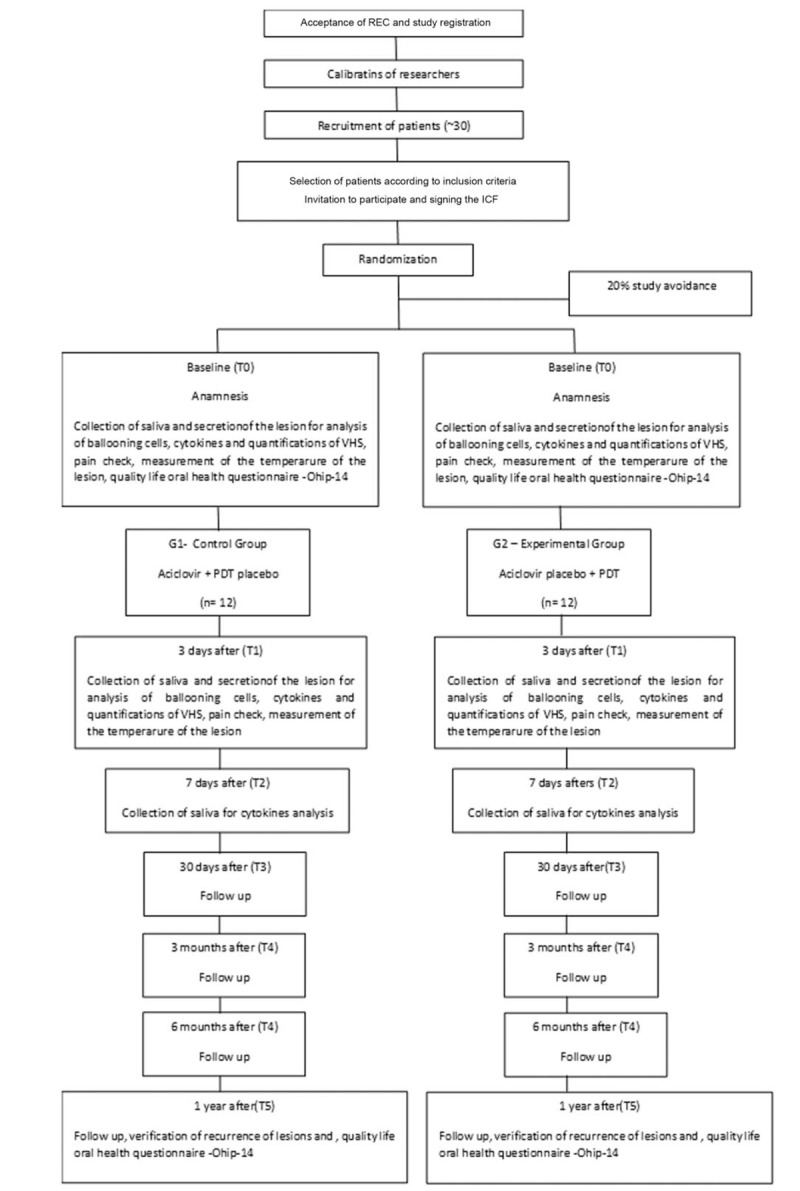
Flowchart. REC = Research Ethics Committee, ICF = Informed Consent Form, OHIP-14 = Oral Health Impact Profile Questionnaire, VHS = virus Herpes Simplex, PDT = photodynamic therapy.

This is a single-center randomized, double-blind, controlled, prospective trial consistent with the criteria for designing a clinical SPIRIT statement. The project will be submitted to the Research Ethics Committee of Universidade Nove de Julho (UNINOVE). After verbal and written explanation of the study, participants who agree to participate in the study will sign the informed consent form (ICF) and the term of consent (TC) for children under 12 to 18 years of age (APM). The study will be conducted in accordance with the Declaration of Helsinki. The project will be registered at www.clinicaltrial.gov. Project receive grant from Brazilian National Council for Scientific and Technological Development - CNPq #165189/2019-3 (CNPq, Portuguese: Conselho Nacional de Desenvolvimento Científico e Tecnológico). Patients will be invited to attend the Dental Clinic of the University of Nove de Julho (UNINOVE), at city of São Paulo, Brazil, from January 2020 to August 2021 for PDT treatment and evaluation. The experimental design will consist of 2 groups. The sample will be composed of healthy patients who have herpes lesions in the vesicle or crust stage. The total time for lesion remission, ballooning cell verification, virus quantification at the site of injury, and saliva will be assessed. Samples (HSV secretion) will be collected at the injury site and saliva for the quantification of inflammatory cytokines. Pain, temperature, and recurrence of the lesion will be assessed at a 1-year follow-up. The OHIP-14 questionnaire will also be used to measure quality of life related to oral health. Patients will return in 3 days (T1), 7 days (T2), 30 days (T3), 6 months (T4), and 1 year (T5) for reevaluation. Every time there is recurrence of the lesion, the clinic will be invited for collection and verification of the lesion site (recurrence in the same place, it will be noted in the labial diagram by sextant) (Fig. 1).

### 2.1 Sample size calculation

The sample was calculated using standard error type 1 of 0.2 and 95% confidence level as indicated in the literature.^[22–27]^ The calculation is consistent, equivalence tests in nonparametric models of survival (Kaplan and Meier, 1958). The survival function, at a given time *t*, can be estimated from observed survival times, relative to the proportion of individuals who survived beyond time *t*. In our case, time *t* is defined as the treatment date, and survival is estimated in days until recovery of the patient. Based on the survival analysis methodology, a sample of 24 participants (12 per group) was calculated. The estimation of differences between groups will be done based on the Kaplan–Meier estimator or limit product estimator.

### 2.2 Evaluators calibration and training

For calibration there will be only 1 examiner (ALSA) who will do 5 pain assessments with visual analogic scale (VAS), which will not be part of the study. There will be 2 evaluations per patient (with a 30-minute interval between them) to check for intraexaminer agreement. The intraclass correlation coefficient (ICC) will be calculated to evaluation will be performed with a 0 to 10-cm millimeter ruler by a researcher that will not be involved in the treatment of the patients. Calibration as described (ICC) shall be performed for all measurable data.

This same examiner will be trained for all evaluations that do not allow quantitative and qualitative measurements such as: collection of secretion and saliva. These procedures are important to maximize the reproducibility of evaluations.

### 2.3 Sample description

The sample will be composed of healthy patients aged 15 to 60 years who have herpes lesions in the prodromal stage, vesicles, ulcers, and crusts.

### 2.4 Inclusion and exclusion criteria

Patients of both sex, with no predilection for race or socioeconomic status, would be included (negative medical history).

Patients with herpes infection in the dry desquamation stage will be excluded. Participants in continuous use of nonsteroidal anti-inflammatory drugs and continued corticosteroid therapy ^[28]^ for <1 week, diabetic participants, smokers who need immunosuppressants, pregnant women and/or nursing mothers, human immunodeficiency virus positive, and hepatitis B or C will be also excluded.

### 2.5 Randomization

To randomly distribute the participants in the experimental groups, a draw with 30 numbers will be made through the program Microsoft Excel, version 2017 (ACRTH). The distribution of groups will be identical (1:1) for the 2 groups. The distribution will be done in a blocking fashion (5 groups of 6 patients). Opaque envelopes will be identified with sequential numbers (1–30) and inside it will be the information of the corresponding experimental group according to the order obtained in the draw. The envelopes will be sealed and will remain sealed in numerical order until the treatment of the lesions. The draw and preparation of the envelopes will be performed by a person not involved in the study. Immediately prior to treatment of the lesions the investigator responsible for the treatment will open 1 envelope (without changing the numerical sequence) and perform the indicated procedure (ALSA). Three more patients will be included in each group in view of the drop out predicted in any clinical study (considering a drop out of 20%). The researcher will be responsible for storing and tabulating the information, and the statistician (EC) will be responsible for analyzing the data obtained at the end of the research. These same envelopes will contain patient records (with anamnesis sheet, ICF, and X-rays) and will be kept on file and will be accessible to patients as soon as they wish.

### 2.6 Study blinding

Only the researcher responsible for performing the treatments (which will open the envelopes of the randomization) will know which treatment was assigned to each patient. The identification of each group will be revealed only after statistical analysis of the data for all those involved in the study by this researcher. Therefore, the researcher responsible for data collection, the microbiologist (APS) and the statistician (EC) will be blind regarding the treatments assigned to the groups. The patient will also be blind to the type of treatment performed, since the treatment of the lesion will be identical in both groups and the treatment with PDT will be simulated in the control group. Aciclovir placebo will be used in the experimental group, so the patient will not know which treatment will be effective.

### 2.7 Pretreatment evaluations

Patients will sign the ICF and the TC, the anamnesis, the OHIP-14 questionnaire, and the data collection injury and questions regarding injury. Saliva and HSV secretions from all patients will also be collected for evaluation of inflammatory cytokines and saliva and HSV secretion for HSV quantification. Treatment will then be performed according to randomization.

### 2.8 Anamnesis

Anamnesis will be performed in both groups. In addition to general patient health questions, demographic data (age, gender, marital status, occupation, educational level, living conditions, and salary), medical history data (primary complaint, current illness status, medical history, dental history, and medications) and will be asked when was the first infection and how many infections occurred within 1 year. The patient will be asked if there is a relationship between stress/begin of the injury and anxiety/begin of the injury.

### 2.9 Experimental draw

Immediately prior to treatment of the herpes lesion the responsible investigator will remove and open 1 envelope (without changing the numerical sequence of the other envelopes) and perform the indicated procedure. In this way, the 24 patients will be allocated in the experimental and control groups (considering a drop out of 20%), as follows:

- G1: Control group (n = 12) – Patients will receive the conventional treatment with acyclovir (described in detail in Section 2.11) and simulation of treatment with PDT (described in detail in Section 2.10).
- G2: Experimental group (n = 12) – Patients will be treated with PDT and will receive a placebo ointment simulating acyclovir cream.

### 2.10 Treatment with photodynamic therapy

In group 2, patients will receive aPDT. The aPDT associates a photosensitive agent with a source of light and oxygen, to reduce the microbial load in places where there is no vesicle present.

The procedures will be performed as described:

1. If the lesions are in the vesicle phase, they will be ruptured with a sterile needle, and the overflow fluid will be cleaned with an absorbent paper taking care not to contaminate adjacent areas. The methylene blue solution at 0.005% concentration (Chimiolux; DMC, São Carlos, SP, Brazil) will be gently placed on the lesions.
2. Application of methylene blue (0.05% Chimiolux 5 [DMC], purified water, and methylene blue) with a carpule syringe and needle (with stop and without bevel) on the lesions.
3. One minute of preirradiation will be expected.^[29]^ The irradiations will be performed with the Laser Duo red laser diode (MMOptics, São Carlos, SP, Brazil) with a wavelength of 660 nm, with a power of 100 mW, energy density of 300 J/cm^2^, with energy of 3 J in the center of the lesion for 30 seconds.^[30]^ The laser head will be positioned in direct contact with the lesion perpendicular to the lesion.^[31]^
4. It will be applied centrally to each isolated lesion that presents with fixed energy per point of 3 J.
5. Wash in abundance with saline (saline solution) until the removal of the photosensitizer is complete.
6. During the laser application both patient and operator will wear specific goggles to eye protection following the international security rules.
7. Patients in group 2 will use placebo ointment as described in the following list.

The patients in group 1 (control-placebo of PDT) will receive an agent with the same vehicle as methylene blue to mimic irrigation with the photosensitizer and the laser will be switched off at the time of application.

The placebo PDT procedures will be performed on the lesion as described:

1. Application of methylene blue placebo with a Carpule syringe and needle (with stop and without bevel) inside the lesions.
2. One minute of preirradiation will be expected.^[29]^
3. The irradiations will be performed with the same device positioned in the same way and at the same time of application; however, the laser will be turned off. The beep sound will be recorded and turned on during application to blind treatment to the patient.
4. It will be applied exactly as described in group 2. It will be washed in abundance with saline (saline solution) until the total removal of the placebo from the photosensitizer.
5. During the simulation of the laser application both patient and operator will wear goggles (Table 1).

**Table 1:**
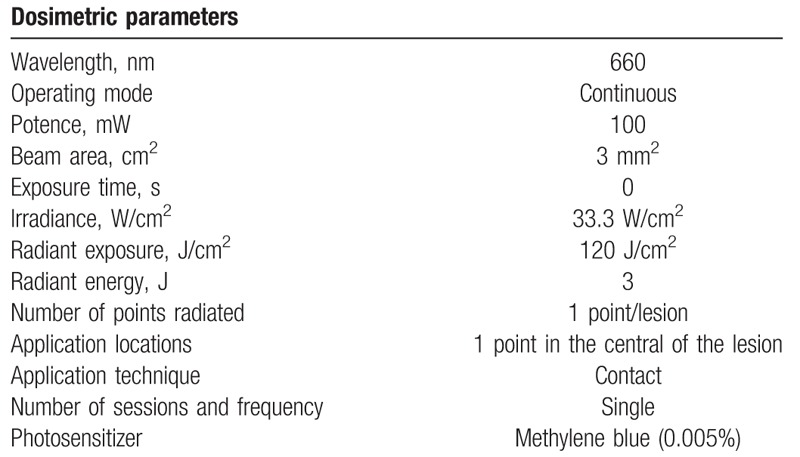
Parameters used in photodynamic therapy.

### 2.11 Treatment with acyclovir

Patients in group 1 (control): Following the simulation of treatment with aPDT, the patient will receive a catheter with acyclovir cream and will be advised to spread on the lesions 4 times a day for 7 days, in which will be their return for reevaluation. The same patient will be reevaluated after 1 month, 3 months, 6 months, and 1 year.

Patients in group G2 (experimental): The patients will perform the aPDT as described and will undergo the same procedures of the patients of group G1, that is, will receive a tube with a placebo cream simulating aciclovir and the same will be advised to spread the cream 4 times per day for 7 days. The patient will return for reassessment after 3 and 7 days and will be followed up after 1 month, 3 months, 6 months, and 1 year for recurrence evaluation.

All treatment proposed in group 1 (simulation of aPDT + delivery of aciclovir cream) in each patient will be concluded in a single session. Treatment for group 2 (aPDT + placebo cream) will also be completed in a single session. All possible adverse effects will be noted and qualified during the treatment protocol and maintenance period (3/3 months) using the questionnaire developed for this protocol. Adverse events will be reported in the results section of the manuscript and will be discussed. The Research Ethics Committee of the Universidade Nove de Julho (UNINOVE) will be notified of any change.

### 2.12 Study variables and time

The primary study variable will be evaluation of the resolution time of the lesions in days (primary objective of the study). For this, a 15-day follow-up will be performed for this variable.

The secondary variables of the study will be

- Follow-up of the recurrences of herpes simplex labialis (HSL) lesion at baseline (T0), 1 (T3), 6 months (T4), and 1 year (T5) follow-up periods.
- Presence of ballooning cells by exfoliative cytology at baseline (T0) and 3 (T1) days after injury.
- Evaluate if the amount of HSV-1 decreases after treatment with aPDT by Quantitative polymerase chain reaction q at the baseline (T0), 3 (T1), and 7 (T2).
- Evaluation of cytokines interleukin (IL)-6, IL-1β, IL-8, tumor necrosis factor alpha (TNF-α), and IL-10 by enzyme-linked immunosorbent assay method in nonstimulated saliva and HSL lesions in the baseline (T0), 3 (T1), 7 (T2), and 30 days after treatment (T3).
- To verify the presence of pain in HSL patients by HSL VAS at baseline (T0), 3 (T1), 7 (T2), and 30 days after treatment (T3).
- Check the temperature at the site of HSL lesion at baseline (T0), 3 (T1), 7 (T2), and 30 days after treatment (T3).
- Quality of life perception of HSL patients related to oral health will be assessed using the OHIP-14 questionnaire at baseline (T0) and 30 (T3) days after treatment.

### 2.13 Detailed study variables

#### 2.13.1 Evaluation of injury resolution time in days

The patients will be followed up in 3 and 7 days and the resolution of the lesion will be monitored until it is completed. The completed will be accompanied by photos and telephone contact and noted in a specific clinical file. For this, a 15-day follow-up will be performed for this variable.^[15]^

#### 2.13.2 Follow-up of HSL injury recurrences

Patients will be monitored at baseline (T0), 1 (T3), 6 (T4), and 1 (T5) follow-up periods. The complete will be accompanied by photos and telephone contact and noted in a specific clinical file. For this, a 1-year follow-up will be performed for this variable. The location of the lesions through sextants on the lips (sextants 1, 2, and 3 to upper left to right and sextants 4, 5, and 6 from right to left, with sextant 2 in the labial filter region and sextant 5 is its lower lip antagonist.

#### 2.13.3 Presence of ballooning cells

Quantify the ballooning cells by means of exfoliative cytology.^[32]^

#### 2.13.4 Collection of ballooning cells for exfoliative cytology

The swab on the lesion of the exfoliative cytology kit of the female swab specimen collection kit (Qiagen, Hilden, Germany) will be gently passed, made the smear on a slide for microscopic examination.

#### 2.13.5 HSV-1 quantification

Collection of HSV lesions secretion: HSV lesion secretion samples will be collected at 1 single site in the central portion of the lesion with sterile swab after treatment of all HSV lesions. They will be stored in 500 μL of Tris-ethylenediaminetetraacetic acid in tubes of 1.5 mL for microcentrifuge (Eppendorf, Hamburg, Germany). During the collection period (in the clinic) the samples will be stored on ice inside a styrofoam. These samples will be properly identified and stored at −80°C (freezer from the UNINOVE Biophotonics Laboratory) until further analysis.

#### 2.13.6 Collection of saliva

Samples of unstimulated saliva (2 mL) will be collected in 50 mL tubes (Falcon tube). In the laboratory 500 μL of pure saliva will be added to 500 μL Tris/EDTA, and stored at −80° C (freezer from the UNINOVE Biophotonics Laboratory).

#### 2.13.7 Extraction of DNA

The DNA extraction will be performed with Meta-G-Nome DNA Isolation - MGN0910 (Epicenter Technologies Corp, Chicago, IL) according to the manufacturer's instructions. For saliva samples and HSV secretion, 100 μL of sample, previously mixed in a Vortex at 12,857*g* (10,000 RPM), 4° C, shall be transferred for 10 minutes to new centrifuge microtubes. The supernatant will be discarded, leaving 25 μL, next to the precipitate. Samples will be mixed in a Vortex for resuspension of the pellet. For each sample a mixture of 2 μL of proteinase K and 300 μL of TCL (tissue and cell lysis), placed in a water bath for 15 minutes and every 5 minutes will be vortexed. After the water bath, the samples will be placed on ice for 5 minutes. About 150 μL of protein precipitation solution reagent and mixed in a Vortex for 5 seconds. It will be centrifuged for 10 minutes, 4°C at 12,857*g* (10,000 RPM). The supernatant will be discarded, leaving only the pellet. About 500 μL of isopropanol will be added and the samples will be poured 40 times. The samples will then be centrifuged again for 10 minutes, at 4°C at 2057*g* (4000 RPM), the samples being poured, removing the isopropanol, leaving only the pellet. About 200 μL of 70% ethanol will be added and centrifuged for 2 minutes, 4°C and 2057*g* (4000 RPM). The ethanol will be withdrawn and the last step will be repeated. The tubes will be opened, inverted to dry, for about 30 minutes. After drying, the resulting pellet will be eluted in Tris/EDTA. After DNA extraction the samples will be stored in a freezer at −20°C and quantified through the spectrophotometer (Nanodrop ND1000; Thermo Fisher Scientific Inc). Prior to polymerase chain reaction (PCR) amplification, a set of primers for HSVP1/P2 corresponding to a DNA sequencing the eight human herpes virus described by Johnson et al (Table 2) will be used. The first HSVP1/P2 amplifies the subtypes HSV-1, HSV-2, Epstein-Barr virus, and cytomegalovirus.^[33,34]^

**Table 2:**
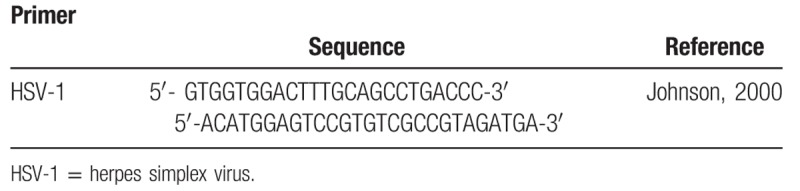
Sequence and reference of primer.

#### 2.13.8 Salivary cytokine profile and HSV lesion secretion

The HSV lesion secretion samples will be collected with sterile swab at 1 site (central lesion site) and stored in 1.5 mL tubes for microcentrifuge (Eppendorf). The collected samples will be stored on ice. These samples will be properly identified and stored at −80°C (UNINOVE Biophotonics Laboratory) until further analysis.

#### 2.13.9 Saliva collect

Samples of unstimulated saliva (2 mL) will be collected in 50 mL tubes (Falcon tube) requesting that the patient spit in a 50-mL centrifuge tube without touching the lips at the edges of the tube on the 1st care. In the laboratory, 1 mL of pure saliva will be stored in 1.5 mL centrifuge tubes at −80°C (freezer from the Biophotonic Laboratory of UNINOVE).^[35]^ Analysis of salivary cytokine profile and HSV lesions: Determination of salivary levels and HSV secretions of the inflammatory markers TNF-α, IL-1β, IL-6, IL-8, and IL-10 will be performed. Cytokine levels will be determined using high sensitivity 8-plex human cytokine by Millipore (Millipore Corporation, Billerica, MA). The miliplex kits will be developed with microspheres and based on immunoassays.

#### 2.13.10 Pain

Pain will be assessed by applying the VAS with a line of 100 mm, with both ends closed. One end has the indication “0” and the other “10” which means respectively no pain and unbearable pain. Instructions for marking will always be given to the patient by the same operator. Each patient will be instructed to mark with a vertical trace the point that best corresponds to the intensity of pain at the time of evaluation.^[36]^

#### 2.13.11 Temperature

The temperature will be measured at the site of the lesion (at its central point) and at its side (healthy skin 2 cm from the edge of the lesion). The local measurement will be measured using the Safety 1st digital thermometer (Safety 1st Montreal Quebec Canada) (Safety 1st, “No Touch Forehead: Xi’an, China,” Columbus, OH).^[36]^

#### 2.13.12 Oral health impact profile (OHIP-14)

This questionnaire is a simplified form of the original OHIP-49 questionnaire,^[37]^ the OHIP-14, and will be used to assess the impact of oral health on the quality of life of the research participants. The OHIP-14 is used to measure perceived needs. It measures the impact of oral changes on oral health related quality of life. The patient responds to 14 questions by assigning to his answers the values 0 (never), 1 (almost never), 2 (sometimes), 3 (most of the time), and 4 (always) (Table 3).

**Table 3:**
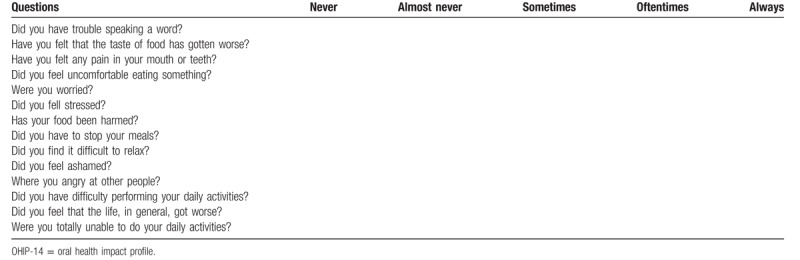
Questions of OHIP-14.

It can be analyzed under 2 optics:

1. Additive method: The points are summed (0–56), with 56 being the greatest impact on quality of life.
2. Domain evaluation: OHIP-14 has a total of 7 domains, which can also be scored from 0 to 4, with 4 being the highest impact. To get the result, add the 2 questions of each domain and divide by 2. Domain 1 refers to functional limitation, domain 2 refers to physical pain, domain 3 refers to physical limitation, domain 4 refers to psychological discomfort, domain 5 refers to psychological inability, social discomfort, and domain 7 refers to general disability (Table 4).

**Table 4:**
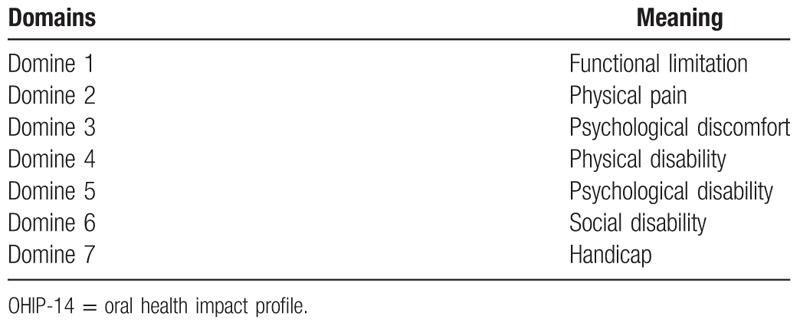
Domains of the OHIP-14 questionnaire.

It will be evaluated at the 1st attendance, after 7 days, if there is any recurrence, and after 1 year the OHIP-14. In the additive method, when the value is >1, the presence of a negative impact on the individual's life may be considered.

The justification for this work is the viral resistance that the continuous use of aciclovir can promote^[7,10]^ and that PDT has shown good results but to date there are no clinical trials, only case reports^[15,20,29]^ and, in addition, PDT can prevent HSL recurrence.^[7,38–40]^

### 2.14 Statistical analysis

Statistical analysis will be performed using only the data obtained from the patients who complete the study. The Kolmogorov–Smirnov test will be applied before the analysis of the normality of each parameter. The *t* test will be used to compare all means. All tests will be performed using Graphpad Prism 5 (Graphpad Software, San Diego, CA). Data will be presented on average and ±standard deviation. The level of significance will be set at *P* < .05.

## 3 Discussion

Oral herpes lesions are very painful, bringing discomfort, and aesthetic impairment.^[6,10]^ The causes of recurrences are uncertain,^[39]^ the gold standard treatment is with antiviral acyclovir,^[9]^ and it is the most commonly used to prevent or suppress VHS^[10,14,40]^ but continued use of this drug may cause high viral resistance.^[7,10]^ Antiviral medications are most effective when the patient uses them for prodromal symptomatology and acyclovir is effective in reducing the duration and symptoms of infection, but the optimal dose is still unknown.^[9]^ Acyclovir may reduce the duration of symptoms but does not prevent recurrence.^[15]^ Because of these factors, aPDT is a good alternative for the treatment of herpetic lesions, which is a very promising treatment modality. Marotti et al^[20]^ in their study with 4 participants in the vesicle phase performed their perforation with a sterile needle to cause the flow of liquid. A 0.01% methylene-blue-soaked cotton ball was placed over the lesion for 5 minutes and the excess photosensitizer was removed. The lesions were irradiated with a wavelength of 660 nm, spot size 0.04 cm^2^ in continuous mode. The power was 40 mW, 2 minutes per point, with energy of 4.8 J (total energy of 19.2 J). After 24 hours, 72 hours, and 1 week, the same wavelength was used changing the parameters, with energy density of 3.8 J/cm^2^, 15 mW, and 0.6 J. No discomfort was reported throughout treatment and follow-up was 6 months with satisfactory results. Ramalho et al^[29]^ reported 2 cases, 1 in a 28-year-old female with a vesicle lesion, in which was punctured, its contents absorbed with absorbent paper, 0.005% methylene blue placed, waiting time of 5 minutes for the photosensitizer to act and the laser applied at a wavelength of 660 nm, with a power of 100 mW, 4.8 J, for 2 seconds per point. The following day, the lesion was already in the crusting phase. The 2nd case reported was from a female participant, 30 years old, who presented in the prodromal or tingle stage, and the participant reported that she had used acyclovir cream 5% when she had prodromal symptomatology. Methylene blue was placed over the macula and the same parameters as in the 1st case were used. The participant was instructed to continue acyclovir cream every 4 hours, and after 24 hours of PDT application, the macula was no longer detectable and herpes lesion did not develop. Marotti et al^[18]^ conducted a study with high intensity laser and PDT. The first case using PDT was in a 23-year-old female with herpetic lesions in the vesicle phase. They were punctured, dried with sterile gauze and 0.001% methylene blue and laser was applied with wavelength 660 nm, with fluency of 100 J/cm^2^, power of 100 mW, 28 seconds, 2.8 J of energy, spot size of 0.028 cm^2^ with total energy of 8.4 J after 24, 48, 72 hours, and 7 days after the 1st application. The parameters used were 20 J/cm^2^, power of 40 mW, 14 seconds per point with energy of 0.56 J per point. The other case was with a 24-year-old male participant using the same parameters as the previous case. Based on these cases and the cases in which Marotti et al^[20]^ used high intensity laser, the authors concluded that PDT has the advantage of low cost. It is based on the interaction of a photosensitizer and laser light in the tissue, causing selective cell damage, generating ROS.^[13]^ The photosensitizer (methylene blue) absorbs visible laser light at an appropriate wavelength (red 660 nm) from the ground state to the singlet state, which has a short lifespan. From this, the photosensitizer can lose energy through nonradioactive processes (phosphorescence) or generate ROS through 2 mechanisms: either by electron transfer, forming free radicals (type I reactions) or by transfer of energy to molecular oxygen forming singlet oxygen (type II reactions).^[21]^ The antimicrobial PDT is an effective alternative to the gold standard treatment, acyclovir, but there are no clinical studies yet to prove the efficacy of PDT in patients with recurrent herpes lesions, only case studies.

This study will be a double-blind clinical trial with the goal of not inducing the researcher who will treat the patient to a positive aPDT result as well as to mask the participant's type of treatment. Blocked randomization was performed so that if there is any interruption of work for any reason, we will have groups with proportional numbers of participants, allowing comparison between them. After approval by the Research Ethics Committee of the Universidade Nove de Julho, patients will be invited to participate in the study by signing the ICF. If the lesions are in the vesicle stage, they will be topically anesthetized with 2% lidocaine gel, as directed by the Research Ethics Committee. Exfoliate cytology of the lesions will be performed as it is a simple and reliable method of confirming HSV infection, according to Barret et al. Microscopically we can observe changes in cytoplasm and nuclear structures. Lange et al,^[41]^ observed that cells exhibit a space between inclusion and the extended “bull's eye” cell membrane. In addition, he observed little frequency of ballooning cells, which are pathognomonic cells in herpes lesions.

Unstimulated saliva collection will be performed before treatment, 3, 7, and 30 days after the procedure, in both groups, to quantify the virus by qPCR and to evaluate the cytokines IL-6, IL-1β, IL-8, TNF-α, and IL-10. For HSV-1 amplification by qPCR, a set of HSVP1/HSVP2 primers will be used, which corresponds to DNA sequencing the eight human herpes virus described by Johnson et al. This primer amplifies HSV-1 subtypes, HSV-2, Epstein-Barr virus, and cytomegalovirus.^[32,33,42]^

In addition, the inflammatory markers TNF-α, IL-1β, IL-6, and IL-8 and IL-10 will be determined by 8-plex high sensitivity human cytokine per Millipore (Millipore Corporation, Billerica, MA), where Marques Filho et al^[43]^ found high levels of these cytokines in the crevicular fluid of patients with peri-implantitis. In the experimental group, methylene blue will be delicately placed and with a 1-minute wait for the photosensitizer to interact with the tissue according to Alvarenga et al.^[43]^ The parameters used for laser application will be performed according to Ramalho et al^[29]^ in case studies reported using 0.005% methylene blue photosensitizer, with the laser head in contact with the lesion, perpendicularly, 3 J energy for 30 seconds, with diode laser at 660 nm wavelength in a single session. Marotti^[20]^ used the laser at the same wavelength, with an energy density of 120 J/cm^2^, with a power of 40 mW, with 2 minutes per point and 4.8 J energy at each point and with 4 points around the lesion in herpes lesions in the vesicle phase, breaking them prior to the application of methylene blue, in which it was used at a concentration of 0.01% (the use of 0.005% is explained based on the large quantity of works with good results). The total energy delivered was 19.1 J. Twenty-four hours after the 1st application, the procedure was repeated, but with energy density of 3.8 J/cm^2^, 15 mW of power, with 0.6 J of energy at the same previously irradiated points, and these parameters were repeated after 72 hours and after a week. There was a decrease in the frequency of vesicle recurrence. De Paula Eduardo et al,^[13]^ found in a literature review that in cases where aPDT was indicated in the vesicle phase, there was a reduction in vesicle title as well as lesion duration. Decreased signs of infection and symptoms are reported within hours of aPDT application, provided they are used at a wavelength around 660 nm and with the appropriate photosensitizer. Based on these studies, we expect a reduction in the time of resolution of lesions, as well as the recurrence of lesions, providing a low cost alternative indicated for patients with viral resistance to the gold standard drug, acyclovir, and, offering an alternative in immunocompromised patients who have a high recurrence of herpetic lesions and in patients who should avoid drug administration by organ impairment where acyclovir is metabolized, as well as in healthy patients who have a high recurrence of lesions, decreasing discomfort.

## Author contributions

**Conceptualization:** Anna Carolina Ratto Tempestini Horliana, Andreia La Selva, Renata Matalon Negreiros, Kristianne Porta Santos Fernandes.

**Data curation:** Renata Matalon Negreiros, Lara Jansiski Motta, Daniella Teixeira Bezerra, Ellen Perim Rosa.

**Formal analysis:** Marina Stella Bello-Silva, Karen Miller Ramalho, Ricardo Scarparo Navarro, Ana Cecília Corrêa Aranha.

**Investigation:** Paulo Henrique Braz-Silva, Anna Carolina Ratto Tempestini Horliana, Sandra Kalil Bussadori.

**Methodology:** Paulo Henrique Braz-Silva, Karen Miller Ramalho, Anna Carolina Ratto Tempestini Horliana.

**Project administration:** Andreia La Selva, Anna Carolina Ratto Tempestini Horliana.

**Resources:** Paulo Henrique Braz-Silva, Sandra Kalil Bussadori, Anna Carolina Ratto Tempestini Horliana.

**Supervision:** Sandra Kalil Bussadori, Kristianne Porta Santos Fernandes.

**Validation:** Anna Carolina Ratto Tempestini Horliana, Kristianne Porta Santos Fernandes.

**Writing – original draft:** Anna Carolina Ratto Tempestini Horliana, Andreia La Selva, Renata Matalon Negreiros.

**Writing – review & editing:** Renata Matalon Negreiros, Anna Carolina Ratto Tempestini, Ana Cecília Corrêa Aranha.

Anna Carolina Ratto Tempestini Horliana orcid: 0000-0003-3476-9064.
